# Ongoing nationwide outbreak of *Salmonella* Agona associated with internationally distributed infant milk products, France, December 2017

**DOI:** 10.2807/1560-7917.ES.2018.23.2.17-00852

**Published:** 2018-01-11

**Authors:** Nathalie Jourdan-da Silva, Laetitia Fabre, Eve Robinson, Nelly Fournet, Athinna Nisavanh, Mathias Bruyand, Alexandra Mailles, Estelle Serre, Magali Ravel, Véronique Guibert, Sylvie Issenhuth-Jeanjean, Charlotte Renaudat, Mathieu Tourdjman, Alexandra Septfons, Henriette de Valk, Simon Le Hello

**Affiliations:** 1 Santé publique France, Saint-Maurice, France; 2 Institut Pasteur, Centre National de Référence des Salmonella, Paris, France; 3 European Programme for Intervention Epidemiology Training (EPIET), European Centre for Disease Prevention and Control (ECDC), Stockholm, Sweden

**Keywords:** *Salmonella* Agona, outbreak, infant, milk powder, international

## Abstract

On 1 December 2017, an outbreak of *Salmonella* Agona infections among infants was identified in France. To date, 37 cases (median age: 4 months) and two further international cases have been confirmed. Five different infant milk products manufactured at one facility were implicated. On 2 and 10 December, the company recalled the implicated products; on 22 December, all products processed at the facility since February 2017. Trace-forward investigations indicated product distribution to 66 countries.

## Identification of the outbreak

At the end of November 2017 the French National Reference Centre (NRC) for *Salmonella* noted an unusual increase in *Salmonella* Agona among infants, with 22 cases identified between August and November 2017 in children younger than six months. On 1 December, interviews with the caregivers of eight of these infants by the French National Institute for Public Health (Santé Publique France (SPF)) identified infant milk products from a single company as a potential source. All but one of the first eight investigated cases had consumed products manufactured at a single facility operated by this company. This facility had previously been associated with an outbreak of *Salmonella* Agona in 2005 [1]. An outbreak investigation is currently ongoing in conjunction with the NRC, SPF, the Ministry of Health and the Ministry of Economy in charge of consumers’ affairs.

## Epidemiological investigations

The operational definition for probable cases as at 11 January 2018 includes children younger than two years with a laboratory-confirmed *Salmonella* Agona infection and with a date of onset of symptoms since 1 January 2017. A confirmed case is a probable case with a *Salmonella* Agona isolate belonging to the outbreak cluster by whole genome sequencing (WGS) or, if WGS has not been performed, an isolate which does not produce H_2_S and gas after 18 h incubation on Kligler iron agar (see *Microbiological investigations* for further details). Cases were excluded if they had a history of travel abroad in the 7 days before symptom onset and probable cases were excluded if after WGS, they were not within the outbreak cluster.

**Figure 1:**
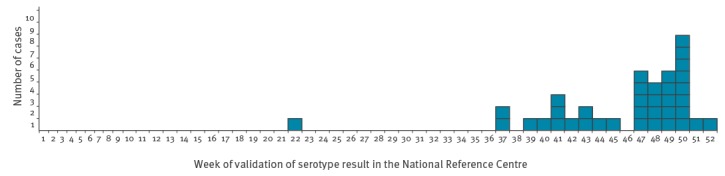
Epicurve of *Salmonella* Agona confirmed cases in infants by week of diagnosis at the National Reference Centre, France, 2017 (n = 37)

As at 11 January 2018, 37 cases have been identified in France, all of whom were confirmed (Figure 1). All cases had gastrointestinal symptoms, and there was no case of bloodstream infection or meningitis. Almost all cases had diarrhoea (34 cases), 23 had bloody diarrhoea, 26 fever and 13 vomiting. One case had symptom onset in April while the remaining cases had onset of symptoms between mid-August and 2 December. The cases included 21 girls and 16 boys. The median age was 4 months (range: 2 weeks–9 months). The cases were scattered across 10 different regions of France.

Among 36 cases whose caregivers have been interviewed to date, 18 were hospitalised for their illness, all of whom have recovered at the time of writing this report. Eleven cases had underlying medical conditions. These conditions were not associated with an increased risk of salmonellosis. However, they may have increased the likelihood of presentation to a healthcare provider and of diagnostic investigations. In the 3 days before symptom onset, 35 of the 36 cases had consumed infant milk products intended for babies aged 0–6 months manufactured by the same company. Five different products have been implicated to date: one product was associated with 26 cases (MILK-A), one product was associated with five cases (MILK-B), one product was associated with two cases (MILK-E) and two products were associated with one case each (MILK-C and MILK-D). All products were manufactured at the same facility. In one confirmed case, exclusive breast-feeding was reported.

No other common foods or drinks were identified among cases. Given the young age of the cases, the majority were still exclusively fed with infant milk products. A variety of types of water (tap and different brands of bottled water) were used to prepare these products. Caregivers were questioned on their practices in relation to the preparation of bottles of milk (e.g. advance preparation, storage, cleaning of equipment) and no issues were identified which would increase the risk of bacterial contamination or bacterial propagation.

## Microbiological investigations

WGS of *Salmonella* isolates has been performed routinely at the French NRC since April 2017. DNA extraction, libraries and high-throughput genome sequencing for our cases were carried out at PIBnet, Institut Pasteur, Paris on the Illumina NextSeq500 platform. Molecular serotyping was done by in-house scripts based on MLST-7, *fliC* and *fliB* gene databases. For all 88 *S*. Agona isolates received in the NRC between 1 January and 31 December 2017, the filtered paired-end reads were aligned with the Agona SL483 reference genome (GenBank accession number NC_011149) [2]. Phylogenetic analysis was performed on the single nucleotide polymorphism (SNP) filtered alignment using the maximum-likelihood method, and the final tree was visualised as previously described [3]. Raw reads of a representative strain are available under the European Nucleotide Archive number ERR2219379 and under Enterobase name SAL_NA11229AA.

**Figure 2:**
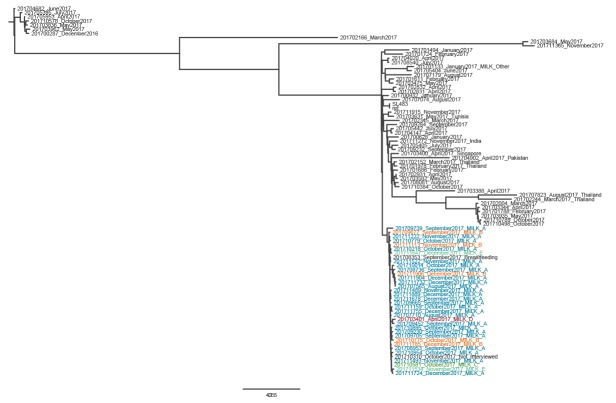
Phylogenetic tree of *Salmonella* Agona strains received at the National Reference Centre, France, 2017 (n = 88)

The phylogenetic analysis of all 88 genomes of *S.* Agona human isolates indicated a unique ST13 type with a high number of 9,519 SNPs in total and revealed that outbreak isolates clustered within a maximum distance of 26 SNPs (Figure 2).

*S*. Agona genomes were mapped against the ST13 SL483 reference strain [2] using Bowtie2 software and filtering low-quality single nucleotide polymorphisms with in-house scripts. Maximum likelihood phylogeny reconstruction was done using IQ-TREE v.1.5.1 with GTR + I + G model with the fast bootstrapping option with 1,000 values. Names include the National Reference Centre identification number, month and year of isolation, source of contamination if provided in patient interview, and travel indication. The tree was rooted on the 201704682 genome.

Interestingly, distinctive microbiological traits were also found in the outbreak strain: it did not produce H_2_S and gas in 18 h incubation on Kligler iron agar. *Salmonella* isolates do typically produce H_2_S and gas in 18 h incubation on Kligler iron agar.

To date, 36 of the 37 confirmed cases belonged to this SNP cluster on WGS. For the remaining case, WGS results are pending but the isolate does not produce H_2_S and gas after 18 h incubation on Kligler iron agar.

## Environmental and product investigations

Environmental and product investigations by the company and the Ministry responsible for consumers’ affairs are still ongoing. However, epidemiological information strongly suggests products manufactured at one facility as the source of infections. The facility manufactures a wide range of infant and toddler foods. Routine microbiological tests of products undertaken by the facility as part of normal production practices were negative. On 2 December 2017, the company voluntarily recalled batches of the three initially implicated products (MILKS-A, B, C) [4]. On 10 December, after parents of one case reported exclusive use of a fourth product (MILK-D), and in light of preliminary results of environmental investigations at the facility, the company recalled all products manufactured at the facility since mid-February 2017 and for which production was associated with one drying tower at the facility [5,6]. On 13 December, five further batches omitted from the recall list issued on 10 December were recalled [7]. On 22 December, as a precautionary measure, the company voluntarily recalled all products manufactured or processed in the facility since February 2017 [6].

## Potential for international spread and international alerts

As at 8 January 2018, trace-forward investigations indicated that recalled products were distributed to 66 countries, including 12 countries in the European Union (EU). An alert was issued on the European Rapid Alert System for Food and Feed (RASFF) on 4 December, and updated as further information on the international distribution network of the products became available [8]. The alert was also issued through the Infosan network operated by the World Health Organization. European public health authorities and microbiologists were alerted about the outbreak on 6 December 2017 by SPF via an *urgent inquiry* for information issued through the Epidemic Intelligence Information System for Food and Waterborne Diseases and Zoonoses hosted by the European Centre for Disease Prevention and Control.

To date, two cases in infants who consumed implicated exported products have been identified in EU countries other than France. The isolate from a case in Spain belonged to the outbreak SNP cluster in WGS performed at the NRC (data not shown in Figure 2). The isolate from a case in Greece did not produce H_2_S or gas after 18 h incubation on Kligler iron agar, and the results of WGS are pending [9].

## Control measures in France

In France, multiple media platforms have been used since 2 December 2017, to inform parents and caregivers about the outbreak, to advise them not to use the implicated products and to recommend appropriate hygiene practices when preparing infant milk products [5]. All recalled products have been published on the website of the French ministry responsible for consumers’ affairs [4-7]. As the initial public alert was issued on a weekend, advice was also given on how to mitigate the risk if alternative products were unavailable over the weekend. In cooperation with the French Society of Paediatrics, advice was issued on alternative infant milk product options [10]. The French Ministry of Health put in place a telephone help service for concerned parents and caregivers. Paediatric and maternity facilities as well as healthcare professionals were also informed.

## Discussion

In France, surveillance of *Salmonella* infections is undertaken by the NRC and SPF. Between 2011 and 2016, *Salmonella* Agona accounted for 2.1% of *Salmonella* isolates identified in infants and received in the NRC, with between eight and 13 isolates received by the NRC a year. The detection of eight isolates of *Salmonella* Agona in infants within a period of 8 days alerted the NRC to the outbreak. No increase in the number of *Salmonella* Agona isolates was observed in other age groups. Prompt investigation provided strong epidemiological evidence pointing to infant milk products manufactured by the same company as the source of the outbreak.

This is the third outbreak of *Salmonella* associated with infant milk products reported in France [1,11]. Similar outbreaks have also been reported elsewhere [12-16]. A *Salmonella* Agona outbreak affecting 141 confirmed cases occurred in France in 2005 and was associated with two different products manufactured within the same facility implicated in the current outbreak [1]. During the 2005 outbreak, samples of the implicated products and environmental samples from the facility yielded isolates with the same PFGE pattern as the clinical isolates. However, only one of 176 and four of 27 samples from the two implicated food products and six of 420 environmental samples tested positive for *Salmonella* Agona, suggesting a low level contamination. The production dates of the positive food samples suggested a persistent environmental contamination. The source of the contamination in the facility was not identified. In the current outbreak, the environmental investigations are still ongoing, and at this stage no obvious source of contamination has been identified within the facility.

The number of cases associated with this outbreak is probably underestimated as cases with mild symptoms may not have consulted a healthcare professional or had comprehensive diagnostic investigations (stool sample, culture, identification of serotype, confirmation at the NRC). Despite this possibility, given the wide distribution of products and the small number of reported cases, the level of contamination of products is probably low. The fact that the current outbreak involves the same serotype as the previous outbreak raises the question whether the organism has persisted in the facility for 12 years. The persistence of *Salmonella* Agona in a dry food production environment in the United States, resulting in two outbreaks 10 years apart, 1998 and 2008, has been reported before [17]. Comparison of human and environmental isolates from 2005 with isolates from the current outbreak is ongoing to investigate this possibility.
